# Functional Dissociation of Confident and Not-Confident Errors in the Spatial Delayed Response Task Demonstrates Impairments in Working Memory Encoding and Maintenance in Schizophrenia

**DOI:** 10.3389/fpsyt.2018.00202

**Published:** 2018-05-29

**Authors:** Jutta S. Mayer, Michael Stäblein, Viola Oertel-Knöchel, Christian J. Fiebach

**Affiliations:** ^1^Department of Psychology, Goethe University, Frankfurt, Germany; ^2^Department of Child and Adolescent Psychiatry, Psychosomatics and Psychotherapy, University Hospital Frankfurt, Goethe University, Frankfurt, Germany; ^3^Department of Psychiatry, Psychosomatic Medicine and Psychotherapy, University Hospital Frankfurt, Goethe University, Frankfurt, Germany; ^4^Brain Imaging Center, Goethe University, Frankfurt, Germany

**Keywords:** schizophrenia, working memory, encoding, maintenance, false memory, spatial

## Abstract

Even though extensively investigated, the nature of working memory (WM) deficits in patients with schizophrenia (PSZ) is not yet fully understood. In particular, the contribution of different WM sub-processes to the severe WM deficit observed in PSZ is a matter of debate. So far, most research has focused on impaired WM maintenance. By analyzing different types of errors in a spatial delayed response task (DRT), we have recently demonstrated that incorrect yet confident responses (which we labeled as false memory errors) rather than incorrect/not-confident responses reflect failures of WM encoding, which was also impaired in PSZ. In the present study, we provide further evidence for a functional dissociation between confident and not-confident errors by manipulating the demands on WM maintenance, i.e., the length over which information has to be maintained in WM. Furthermore, we investigate whether these functionally distinguishable WM processes are impaired in PSZ. Twenty-four PSZ and 24 demographically matched healthy controls (HC) performed a spatial DRT in which the length of the delay period was varied between 1, 2, 4, and 6 s. In each trial, participants also rated their level of response confidence. Across both groups, longer delays led to increased rates of incorrect/not-confident responses, while incorrect/confident responses were not affected by delay length. This functional dissociation provides additional support for our proposal that false memory errors (i.e., confident errors) reflect problems at the level of WM encoding, while not-confident errors reflect failures of WM maintenance. Schizophrenic patients showed increased numbers of both confident and not-confident errors, suggesting that both sub-processes of WM—encoding and maintenance—are impaired in schizophrenia. Combined with the delay length-dependent functional dissociation, we propose that these impairments in schizophrenic patients are functionally distinguishable.

## Introduction

Impairments in working memory (WM) are considered a core cognitive deficit in schizophrenia (1, 2) with significant consequences on the patients' functional outcomes (3, 4). Even though extensively investigated, the nature of WM deficits in patients with schizophrenia (PSZ) is not yet fully understood. In particular, it is a matter of debate which specific component processes of WM are responsible for the performance deficits observed in the majority of PSZ.

The delayed response task (DRT) has been widely used to study the cognitive and neurophysiological underpinnings of spatial WM (5, 6). More recently, it has also been used for neurocomputational modeling (7, 8). The prototypical DRT involves the presentation of a stimulus, followed by a short delay during which the stimulus is no longer presented but has to be actively maintained in WM. Finally, a probe is presented, often involving two response choices of which one matches the original memory stimulus (1, 9, 10). The DRT, accordingly, can be subdivided into three clearly demarcated phases of (i) encoding information into WM during the presentation of the stimuli, (ii) active maintenance of those memoranda during the delay phase, and (iii) retrieval of WM contents for purposes of generating a behavioral response during the probe phase (10). It is therefore well suited for studying WM component processes and their dysfunctions in schizophrenia (1, 10). Most research to date focused on impairments in WM maintenance (9–12) which have been associated with a prefrontal dysfunction in terms of altered activation (1, 13–18) and synaptic transmission (19–22).

Other research, however, suggests that abnormal WM encoding might be the primary reason underlying the severe WM deficit in schizophrenia (2). In line with this encoding hypothesis, it has been demonstrated that the perceptual encoding of information and its transfer into a more durable WM representation is slower (23–27) and sometimes less precise in PSZ (28–30). Encoding problems may also arise from a selective impairment of top-down attentional control needed to select relevant and to ignore irrelevant items (31, 32). Moreover, electrophysiological evidence (33–39) and functional magnetic resonance imaging (fMRI) studies (18, 27, 40, 41) provide additional support for a potentially primary role of early-stage visual processing and/or higher-level cognitive processes for abnormal WM encoding in schizophrenia.

One issue that complicates the investigation of encoding and maintenance processes—both in healthy controls (HC) and PSZ—is the difficulty of dissociating these processes in behavioral paradigms. In the classical DRT that has been widely used to assess maintenance deficits, response accuracy and reaction time (RT) are recorded at the end of each trial and therefore are compound measures that may be influenced by cognitive processes from all three task phases. In order to isolate WM encoding processes in the DRT, we previously introduced a novel approach based on the analysis of different types of erroneous responses depending on the trial-to-trial level of self-reported subjective response confidence (42–44). Specifically, we reasoned that incorrect responses that were however given with confidence (which we label as false memory errors) most likely reflect a problem at the encoding stage, i.e., despite erroneous encoding, successful maintenance nevertheless leads to a high confidence rating. In contrast, incorrect/not-confident responses are more likely caused by the degradation of representations during the active maintenance of WM contents, resulting in judgments of low confidence. In line with the encoding hypothesis, we demonstrated that the percentage of incorrect/confident responses (i.e., false memory errors) in HC in a spatial DRT decreased when the processes that support WM encoding were facilitated, while PSZ did not benefit from this facilitation effect (42, 43). In addition, the rate of false memory errors was increased in the spatial DRT in PSZ and their non-affected first-degree relatives compared to HC and psychiatric controls, providing further evidence for impaired WM encoding (44).

In our previous studies we had applied this paradigm to investigate cognitive processes during the encoding phase and their impairments in schizophrenia (42–44). However, while it is theoretically highly plausible to associate not-confident errors with processes of WM maintenance, this second assumption of our approach has so far not been explored systematically. To fill this gap, and to more explicitly dissociate the cognitive processes with which confident vs. not-confident errors are associated, we here varied the length of the delay period in a spatial DRT between 1, 2, 4, and 6 s. We hypothesized that increasing the time over which visual-spatial information has to be maintained in WM differentially affects the percentages of false memory (incorrect/confident) vs. incorrect/not-confident responses. Specifically, if incorrect/not-confident errors are indeed related to processes of WM maintenance, their occurrence should increase with longer delay lengths, reflecting the increased difficulty of maintaining visual-spatial representations over longer periods of time. In contrast, we predict that the percentage of incorrect/confident responses should not vary as a function of delay length, which would support our hypothesis that false memory errors are not primarily caused by a loss of the spatial representation during maintenance, but rather reflect difficulties during WM encoding.

We also included PSZ in this study: On the one hand, we sought to replicate our previous finding of increased false memory responses in schizophrenia with this new variant of the DRT including different delay lengths. Specifically, given our previous evidence that problems during WM encoding contributed to WM deficits in schizophrenia, we expected that the percentage of confident (i.e., false memory) errors would be increased in PSZ compared to HC independent of delay length. In addition, we tested if deficits in WM maintenance contributed to WM impairments in schizophrenia as well. In this case, the percentage of incorrect/not-confident responses should be increased in PSZ compared to HC, and should more strongly increase with longer delay lengths in PSZ than in HC.

## Materials and methods

### Participants

Twenty-four PSZ and 24 demographically matched HC participated in this study. All patients met diagnostic criteria for schizophrenia (22 paranoid, 2 residual) according to the Diagnostic and Statistical Manual of Mental Disorders, Fifth Edition (45). All patients were inpatients treated at the Department of Psychiatry, Psychosomatic Medicine and Psychotherapy of the University Hospital Frankfurt/Main, Germany. The German language versions of the SCID-I and SCID-II (46) were carried out with all participants by a trained clinical psychologist to confirm the diagnosis, to rule out any comorbidities in the patient group, and to assure that no control subject was suffering from a mental disorder or personality disorder. Demographic and clinical information are summarized in Table 1. The groups were matched on age [*t*_(46)_ = −0.59, *p* = 0.56], gender [χ^2^_(1, *N* = 48)_ = 0.82, *p* = 0.37], premorbid IQ [*U* = 205.5, *p* = 0.19]^1^, and handedness [χ^2^_(1, *N* = 48)_ = 1.02, *p* = 0.31]. All PSZ and HC were European-Caucasians. There was a significant group difference in years of education [*t*_(46)_ = 3.27, *p* < 0.01], which presumably reflects the effects of schizophrenia on educational attainment rather than a premorbid demographic difference. Years of parental education, however, did not differ between groups [mother, *t*_(38)_ = 0.71, *p* = 0.49; father, *t*_(36)_ = 1.22, *p* = 0.23]. Note that missing data (here: concerning IQ and parental education) were handled by excluding cases analysis by analysis (see Table 1).

**Table 1:** Demographicand clinical information.

	**PSZ**	**HC**
	***n*** = **24**	***n*** = **24**
Age	40.67 (11.65)	38.88 (9.66)
Range	21–59	24–60
Female/male	7/17	10/14
Race (Caucasian)	24	24
Handedness (right/left)	23/1	24/0
Years of education	14.83 (2.48)	17.63 (3.41)
Years of education		
Mother^a^	11.40 (4.62)	12.30 (3.36)
Father^b^	12.22 (4.35)	13.95 (4.41)
IQ^c^	104.00 (11.98)	108.43 (11.97)
CPE^d^, mg/day	501.13 (265.57)	n/a
Years of illness	10.83 (8.41)	n/a
PANSS^e^–positive	14.92 (4.27)	n/a
SAPS^f^	20.93	
PANSS—negative	16.17 (6.31)	n/a
SANS^g^	25.58	
PANSS—general	31.29 (7.92)	n/a

*Mean values are shown. SD is given in parenthesis. PSZ, patients with schizophrenia; HC, healthy controls*.

a * Four patients and four controls could not provide this information*.

b * Six patients and four controls could not provide this information*.

c * measured with the MWT-B (Mehrfachwahl-Wortschatz-Intelligenztest 47; the German version of the National Adult Reading Test). Data from one control subject and one patient were not included due to insufficient German language skills*.

d * CPE, Chlorpromazine equivalent; CPEs were calculated based on Andreasen et al. (48). One patient was not medicated. Data from one patient treated with Flupentixol and Amisulpride were not included. One patient also received an antidepressant and one patient an antidepressant in combination with a benzodiazepine*.

e * PANSS, Positive and Negative Symptom Scale*.

f * SAPS, Scale for the Assessment of Positive Symptoms*.

g * SANS, Scale for the Assessment of Negative Symptoms. PANSS scores were converted into SAPS and SANS scores based on van Erp et al. (49)*.

It was ensured that all patients were in a stable clinical condition after a psychotic episode by applying the following criteria: symptoms and symptom severity had to be consistent for at least 2 weeks before assessment; attention and vigilance had to be sufficient to take part in the study. All but one patient were medicated with a second-generation antipsychotic, and one patient also received a first-generation antipsychotic (see Table 1 for more details). It was assured that the last change in medication dosage was made not <2 weeks before assessment. The current daily Chlorpromazine equivalent (CPE)—representing the amount of a given typical or atypical antipsychotic drug equivalent to 100 mg chlorpromazine—was determined for each patient based on the empirically derived conversion coefficients reported by Andreasen et al. (48). The time an individual has been on a given dose was not taken into account when calculating CPEs. Symptom severity of PSZ was rated with the Positive and Negative Syndrome Scale (PANSS) (50) by a trained clinical psychologist. HC were recruited from the community, had no history of DSM-5 Axis 1 or Axis 2 disorders, and were medication-free. It was assured that none of the control subjects had a family history of schizophrenia or any other major mental disorder, using a semi-structured interview. To rule out schizotypal personality, HC were screened using the Schizotypal Personality Questionnaire (SPQ) (51). No control participant scored high on the SPQ (*M* = 9.13, *SD* = 6.12, range: 0–21, highest possible value: 74). All subjects had normal or corrected-to-normal vision. Common exclusion criteria for both groups were a history of head injury, neurological disorder, or current substance abuse. The study protocol was approved by the ethical board of the medical department of Goethe University, Frankfurt/Main, Germany. All subjects gave written informed consent in accordance with the Declaration of Helsinki and were paid for their participation.

### Stimuli, task, and procedure

**Figure 1:**
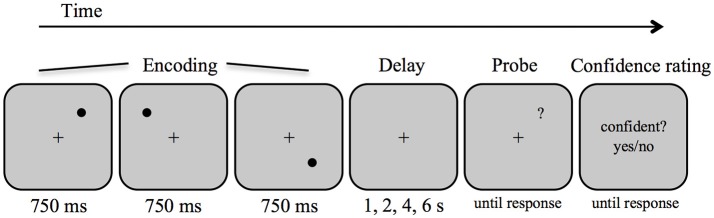
Schematic diagram of the procedure and stimuli in the spatial delayed response task.

Stimuli were presented and responses collected on a PC running Matlab software (Mathwork Inc., Natick, USA) and the Psychophysics Toolbox (52). Target stimuli were black circles of approximately 0.48° visual angle diameter, displayed on a white background (see Figure 1). Stimuli could occur on 16 positions spaced evenly apart (1.9°) along an imaginary circle (4.8° radius) around a centrally presented fixation cross (0.36° width). The positions of 0°, 90°, 180°, and 270° were excluded. Within each trial, the target positions were determined pseudo-randomly with the constraint that the targets appeared in three different quadrants of the screen and that they appeared at least two positions (3.8°) apart from each other on the imaginary circle.

Each trial began with the presentation of the fixation cross for 1 s, then three circles were presented sequentially at three different positions, each for a duration of 750 ms and separated by an inter-stimulus interval of 250 ms. After a variable delay interval (1, 2, 4, or 6 s), a question mark (0.48° visual angle) was presented as a probe until a response was given. Participants indicated whether the position of the question mark matched one of the target positions by a left or right key press for match and non-match, respectively. Half of the trials were matches. In the non-match trials, the question mark always appeared at a neighboring position of one of the targets (i.e., displacement of 1.9°), to hold response difficulty constant. Participants made the response with the index finger and the middle finger of their dominant hand and were instructed to respond as fast and accurately as possible. Immediately after the decision, participants indicated how confident they were in their response by making a non-speeded response for confident vs. not confident using two additional buttons on the keyboard. An inter-trial interval of 3 s followed. Participants performed one practice block (10 trials) followed by four experimental blocks of 32 trials each. There were 32 trials for each delay length, which were randomized across the four blocks.

### Analyses

We calculated mean accuracy, mean RTs, and the percentages of incorrect/confident and incorrect/not-confident responses. In addition we used Signal Detection Theory (53, 54) and calculated d′ scores as measure of signal detection sensitivity, defined as d′ = z(hit rate) – z(false alarm rate). Response bias was calculated as c = −0.5 ^*^ (z(hit rate) + z(false alarm)) (54). Extreme hit and false alarm rates were adjusted by replacing rates of 0 with 0.5/*n*, and rates of 1 with (*n* – 0.5)/*n*, where n is the number of signal trials (i.e., match) or noise trials (i.e., non-match).

Repeated-measures ANOVAs were conducted to test whether WM performance indices (RT, accuracy, d′, c) and the percentage of specific error types (i.e., incorrect/confident vs. incorrect/not-confident responses) varied as a function of delay length (1, 2, 4, 6 s) and differed between groups (PSZ vs. HC). For each group and each factor level, dependent variables were assessed for normality using the Shapiro-Wilk-Test. Homogeneity of error variances across groups was assessed using Levene's test and homogeneity of covariances were assessed by Box's test. These results are reported in the Supplementary Materials (see Supplementary Material 1.1.) In case of violation of any of these assumptions, we reported in the Supplementary Materials additionally re-analyses of the respective effects with non-parametric tests (see Supplementary Material 1.1). Greenhouse–Geisser corrected *p*-values were reported in cases where ANOVA sphericity assumptions were violated, which was determined with a Mauchly's test for sphericity (*p* < 0.05). To resolve significant main and interaction effects, separate one-way ANOVAs or *t*-tests (two-tailed) were computed, which were Bonferroni corrected for multiple comparisons. Homogeneity of error variances across groups was assessed with Levene's test when examining group differences using *t*-tests. In cases where homogeneity of error variances was violated, *p*-values not assuming equal variances were reported. Furthermore, we included trend analyses testing for linear and non-linear (i.e., quadratic and cubic) trends in the data.

To explore possible relationships between performance indices (accuracy, RT, d′, and c) as well as the percentages of error types (incorrect/not-confident responses, incorrect/confident responses) and symptoms as well as medication, these indices were correlated, across different delay conditions, with ratings of positive and negative symptoms and the daily CPE dose using Pearson's correlation coefficient. Because dose equivalence coefficients were not available for Flupentixol and Amisulpride (48), data of one patient treated with these drugs were not included in correlational analyses including CPE dose.

For all analyses, missing data were handled by excluding cases analysis by analysis.

## Results

### Accuracy and reaction times

**Figure 2:**
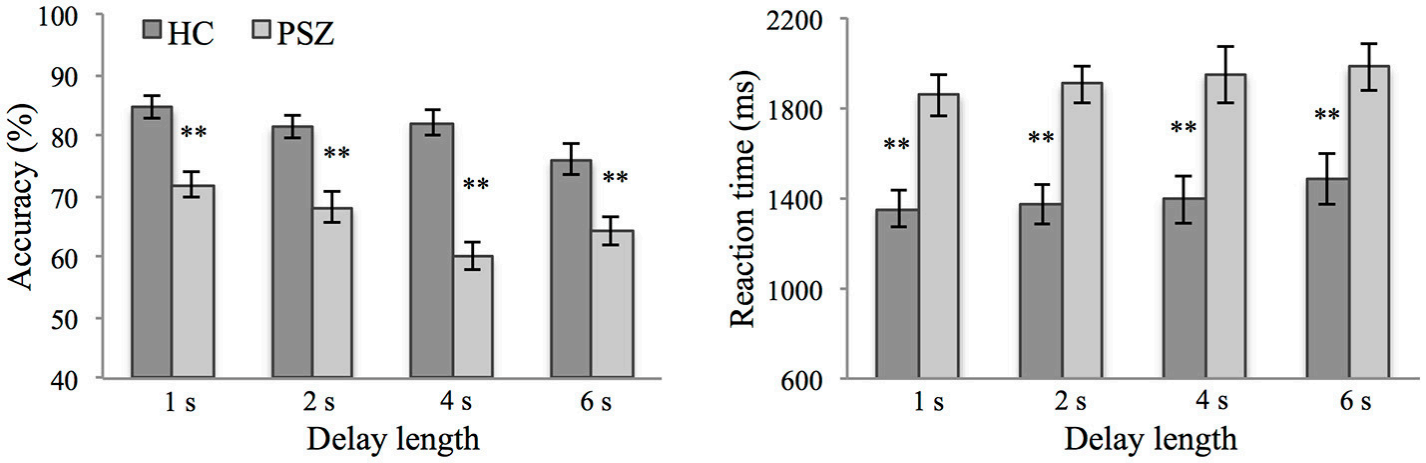
WM accuracy and reaction times at different delay lengths in patients with schizophrenia (PSZ) and healthy controls (HC). Error bars represent the standard error of the mean. Significant group differences at different delay lengths are marked. **indicates *p* < 0.01, corrected for multiple comparisons. Error variances were equal across groups for all delay lengths (Levene's test, *p* > 0.05).

Response accuracy and RTs are shown in Figure 2. 2 × 4 repeated-measures ANOVA were conducted to test for differences in accuracy and RTs as a function of delay length (1, 2, 4, 6 s) and group (PSZ vs. HC) (see Supplementary Material 1.1 for a description of the fulfillment of the ANOVA's assumptions).

As expected, response accuracy was significantly lower in PSZ compared to HC [*F*_(1, 46)_ = 33.20, *p* < 0.001, η^2^ = 0.42]. Response accuracy decreased with longer delay lengths [*F*_(3, 138)_ = 13.20, *p* < 0.001, η^2^ = 0.22] and was explained best by a linear trend [*F*_(1, 46)_ = 30.42, *p* < 0.001, η^2^ = 0.40; quadratic trend, *F*_(1, 46)_ = 1.90, *p* = 0.175; cubic trend, *F*_(1, 46)_ = 0.43, *p* = 0.515]. As indicated by a significant interaction between the factors delay length and group [*F*_(3, 138)_ = 5.45, *p* < 0.01, η^2^ = 0.11], this delay-dependent decrease in accuracy was stronger in PSZ [*F*_(3, 69)_ = 11.05, *p* < 0.001, η^2^ = 0.32] than HC [*F*_(3, 69)_ = 7.11, *p* < 0.001, η^2^ = 0.24]. RTs were significantly longer in PSZ compared to HC [*F*_(1, 46)_ = 15.44, *p* < 0.001, η^2^ = 0.25], and increased with longer delay lengths [*F*_(2.48, 114.04)_ = 2.83, *p* = 0.052, η^2^ = 0.06]. This delay-dependent increase followed a linear trend [*F*_(1, 46)_ = 7.22, *p* < 0.05, η^2^ = 0.14] rather than a quadratic [*F*_(1, 46)_ = 0.203, *p* = 0.654] or cubic trend [*F*_(1, 46)_ = 0.018, *p* = 0.894] and did not differ between groups [interaction effect: *F*_(2.48, 114.04)_ = 0.12, *p* = 0.93].

### Confident and not-confident error responses

A 2 × 4 × 2 repeated-measures ANOVA was conducted to test for differences in the percentage of type of error response (confident vs. not confident) as a function of delay length (1, 2, 4, 6 s) and group (PSZ vs. HC) (see Supplementary Material 1.1 for a description of the fulfillment of the ANOVA's assumptions). This analysis revealed a significant interaction between the factors error type and delay length [*F*_(3, 138)_ = 4.78, *p* < 0.01, η^2^ = 0.09], indicating that across participants the percentage of incorrect/not-confident responses increased with longer delay lengths [*F*_(3, 141)_ = 13.38, *p* < 0.001, η^2^ = 0.22] and was explained best by a linear trend [*F*_(1, 47)_ = 31.83, *p* < 0.001, η^2^ = 0.40; quadratic trend, *F*_(1, 47)_ = 1.78, *p* = 0.189; cubic trend, *F*_(1, 47)_ = 0.00, *p* = 0.986]. In contrast, the percentage of incorrect/confident responses did not vary between delay lengths [*F*_(3, 141)_ = 0.39, *p* = 0.76].

When comparing the WM deficit of PSZ with performance in the control group, the ANOVA revealed a significant group effect [*F*_(1, 46)_ = 33.20, *p* < 0.001, η^2^ = 0.42 (see also results for response accuracy in the previous section)], but no interaction between the factors group and error type [*F*_(1, 46)_ = 0.09, *p* = 0.77]. Thus, both, the percentage of incorrect/confident responses [ = false memory errors, 18.95% (SD = 10.48) vs. 11.01% (SD = 6.94) for PSZ and HC, respectively, *t*_(46)_ = −3.09, *p* < 0.01] and the percentage of incorrect/not-confident responses [14.89% (SD = 9.43) vs. 8.01% (SD = 4.73) for PSZ and HC, respectively, *t*_(33.87)_ = −3.19, *p* < 0.01], were increased in PSZ compared to HC.

**Figure 3:**
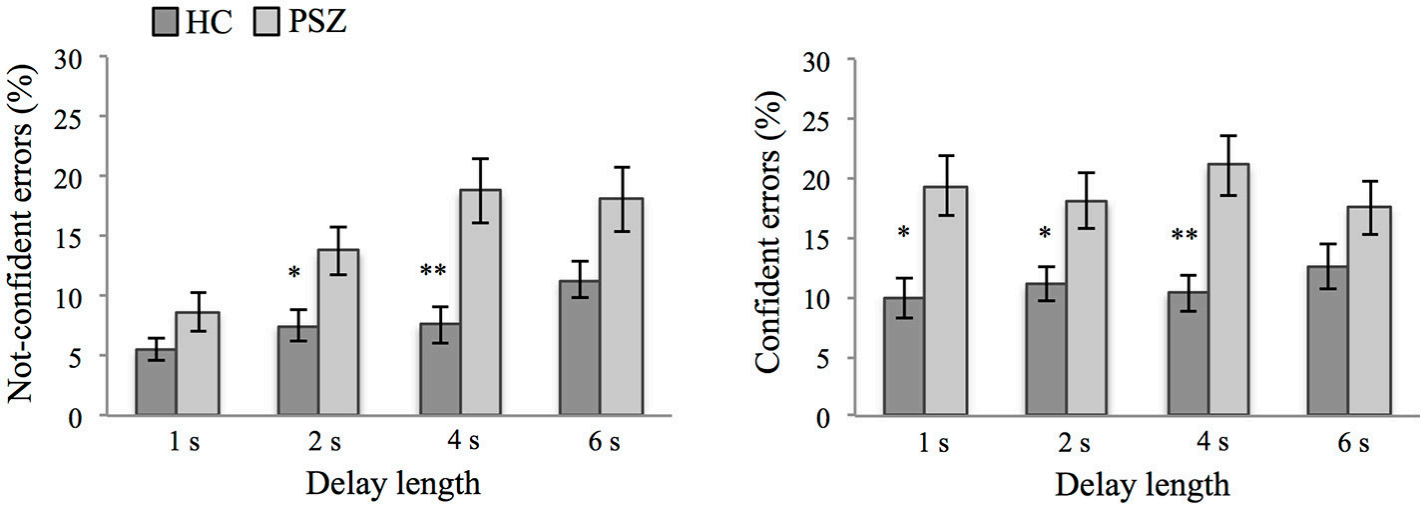
Type of errors. Not-confident errors and confident errors at different delay lengths in patients with schizophrenia (PSZ) and healthy controls (HC). Error bars represent the standard error of the mean. Significant group differences at different delay lengths are marked. * indicates *p* < 0.05 and ** indicates *p* < 0.01, corrected for multiple comparisons. For confident errors, error variances were not equal across groups for delay lengths of 1 s, 2 s, and 4 s (Levene's test, *p* < 0.05). For not-confident errors, error variances were not equal across groups for delay lengths of 1 s, 4 s, and 6 s (Levene's test, *p* < 0.05).

Finally, the three-way interaction between the factors delay length, response type, and group was not significant [*F*_(3, 138)_ = 1.68, *p* = 0.17], indicating that in the present sample the differential effect of delay length on the amount of confident errors (i.e., not delay-dependent; false memory errors) and not-confident errors (i.e., delay-dependent) did not significantly differ between groups (see Figure 3). Across types of errors, however, the effect of delay length was stronger in PSZ compared to HC [significant interaction between the factors delay length and group, *F*_(3, 138)_ = 5.45, *p* < 0.01, η^2^ = 0.11, see also response accuracy].

### Sensitivity (d′) and response criterion (c)

2 × 4 repeated-measures ANOVA were conducted to test for differences in sensitivity and the response criterion as a function of delay length (1, 2, 4, 6 s) and group (PSZ vs. HC) (see Supplementary Material 1.1 for a description of the fulfillment of the ANOVA's assumptions).

**Figure 4:**
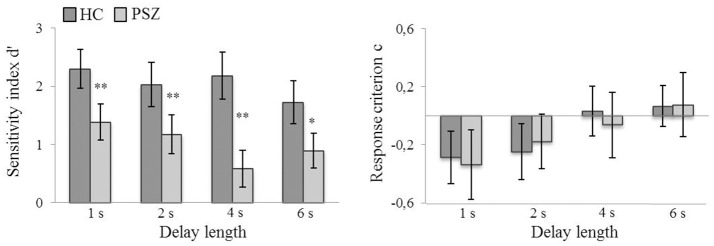
Sensitivity (d′) and response criterion (c) at different delay lengths in patients with schizophrenia (PSZ) and healthy controls (HC). Error bars represent the standard error of the mean. Significant group differences at different delay lengths are marked. *indicates *p* < 0.05 and **indicates *p* < 0.01, corrected for multiple comparisons. Error variances were equal across groups for all delay lengths (Levene's test, *p* > 0.05). Participants' response strategy changed from more liberal (i.e., negative values signify a bias toward responding match) to more conservative (i.e., positive values signify a bias toward responding non-match) with longer delay lengths.

Because the daily CPE dose correlated with d′ (*r* = 0.42, *p* < 0.05, see section Correlations with medication status), individual CPE dose was included as a covariate in the ANOVA. The sensitivity of the discrimination between target and non-target position in the DRT (as determined using Signal Detection Theory sensitivity index d′) was significantly lower in PSZ compared to HC [*F*_(1, 44)_ = 26.36, *p* < 0.001, η^2^ = 0.38]. Furthermore, d′ decreased with increasing delay lengths [*F*_(3, 132)_ = 3.62, *p* < 0.05, η^2^ = 0.08] and was explained best by a linear trend [*F*_(1, 44)_ = 10.71, *p* < 0.01, η^2^ = 0.20; quadratic trend, *F*_(1, 44)_ = 1.74, *p* = 0.19; cubic trend, *F*_(1, 44)_ = 0.25, *p* = 0.62]. As indicated by a significant interaction between the factors delay length and group [*F*_(3, 132)_ = 2.83, *p* < 0.05, η^2^ = 0.06], this delay-dependent decrease was only slightly stronger in PSZ [*F*_(3, 63)_ = 3.61, *p* < 0.05, η^2^ = 0.15], than HC [*F*_(3, 69)_ = 3.15, *p* < 0.05, η^2^ = 0.12] (see Figure 4).

The response criterion c also increased with longer delay lengths [*F*_(2.61, 120.15)_ = 13.40, *p* < 0.001, η^2^ = 0.23] and was also explained best by a linear trend [*F*_(1, 46)_ = 32.74, *p* < 0.001, η^2^ = 0.42; quadratic trend, *F*_(1, 46)_ = 0.023, *p* = 0.88; cubic trend, *F*_(1, 46)_ = 0.95, *p* = 0.34]]. However, there was neither a significant group effect [*F*_(1, 46)_ = 0.011, *p* = 0.92], nor a significant interaction between the factors delay length and group [*F*_(2.61, 120.15)_ = 0.55, *p* = 0.62] on the response criterion c (see Figure 4). Thus, when increasing the delay length, participants' response strategy changed from more liberal to more conservative but, importantly, PSZ and HC did not differ in their response bias.

### Analyses with subgroups matched on education

To rule out the possibility that the observed effects on performance indices and the percentages of percentages of errors types were a consequence of group differences in education, we re-ran the same analyses with a subgroup of PSZ and HC who were matched on the number of years of education (*N* = 18 per group). The results were highly comparable to the results found in the analysis of the entire sample (see Supplementary Material 1.2).

### Correlational analyses

#### Correlations with medication status

In PSZ, there was a positive correlation between the daily CPE dose and the sensitivity index d′ (*r* = 0.42, *p* < 0.05). None of the other performance indices (accuracy, RT, c) nor the percentages of error types (incorrect/not-confident responses, incorrect/confident responses) correlated significantly with medication status (all *p*-values > 0.10; for details see Supplementary Material 2, Table S6).

#### Correlations with symptom severity

None of the performance indices (accuracy, RT, d′, and c) correlated with the severity of positive nor negative symptoms. The percentages of confident and not-confident error responses also showed no correlations with symptom severity (all *p*-values >0.24; for details see Supplementary Material 2, Table S7).

## Discussion

### Functional dissociation of confident and not-confident errors in the DRT

The present study provides important insights into the mechanisms that contribute to failures in WM by analyzing different types or errors in the spatial DRT task in healthy persons as well as PSZ. Across both groups, we demonstrate that increasing the length of the delay period of the DRT had differential effects on the amount of confident vs. not-confident errors, which supports our proposal of a functional dissociation between these types of errors in the spatial DRT. Specifically, the amount of incorrect/not-confident responses increased linearly with longer delay lengths, suggesting that this type of error reflects difficulties in the process of maintaining spatial representations in WM. In contrast, the amount of incorrect/confident responses did not vary with delay length, which is consistent with our hypothesis that confident errors are not primarily caused by a loss of the memory representation during the delay period. Consistent with our previous reports in HC and PSZ (42–44, 55) these findings provide further evidence that false memory errors in the spatial DRT most likely reflect difficulties of WM encoding whereas not-confident errors reflect difficulties of WM maintenance.

### WM deficits in schizophrenia

WM deficits are a hallmark of schizophrenia (1, 2, 10). Spatial WM performance as assessed in terms of accuracy, RTs, and sensitivity (d′) was markedly reduced in our patient sample compared to HC. Moreover, WM accuracy and d′ decreased with longer delay lengths, and the delay-dependent decrease in accuracy was stronger in PSZ than HC. Thus, with increased demands on mnemonic processes required during the maintenance phase, the patients' deficit became more pronounced, which is in line with previous reports of a maintenance deficit in spatial WM (9, 11, 12, 23, 25, 30, 56). Interestingly, WM performance reached a minimum at a delay length of 4 s and did not further decrease at a delay length of 6 s in PSZ. We cannot exclude that the delay length of our longest delay condition, i.e., 6 s was too short, relative to the 4 s delay, to detect further maintenance deficits. However, a very similar pattern, i.e., no further performance decline in PSZ when the delay phase was increased from 4 to 8 s, was reported by Lencz et al. (30) in a visuo-spatial WM task with spatially complex patterns, which provides converging evidence for our result. Together these findings might point to a faster decline in the ability to actively maintain information in spatial WM in schizophrenia—a hypothesis that calls for further investigation with larger sample sizes (see also Limitations).

### False memory errors reflect WM encoding deficits in schizophrenia

Deficits on complex measures such as accuracy or sensitivity can stem from various sources of cognitive impairment. For example, the pattern of a faster decline with increasing delay lengths might also, in principle, result from poorer encoding processes. Accordingly, in order to determine the degree to which different WM component processes (i.e., encoding vs. maintenance) contribute to the deficit in schizophrenia, the present study analyzed different types of errors in the DRT in PSZ vs. HC. Specifically, the amount of confident errors was assessed as an indicator of difficulties during WM encoding, whereas not-confident errors were assessed as an indicator of failures of WM maintenance.

The present study replicates previous reports of increased rates of confident yet incorrect responses in the DRT in PSZ compared to HC (43, 44, 55). Given that confident errors most likely represent failures of WM encoding, the increased rate of false memory errors observed in the present patient sample suggests that encoding failures contribute to the severe WM deficit in schizophrenia—a conclusion that provides converging evidence for results derived from clinical studies that examined different encoding conditions rather than response types (2, 23, 24, 26, 30, 31, 57).

### Not-confident errors represent failures of WM maintenance in schizophrenia

In the present study, PSZ not only showed a higher rate of confident errors but also a higher rate of not-confident errors than HC. Given that not-confident errors most likely reflect a failure of WM maintenance, this finding suggests that failures of maintenance contribute to the WM deficit as well, which is consistent with the storage deficit reported in previous studies on spatial WM (9, 11, 12, 25, 30, 56, 58, 59). However, it is important to note that the differential effect of delay length on the amount of not-confident (i.e., delay-dependent) and confident (i.e., not delay-dependent) errors did not significantly differ between PSZ and HC. In other words, whereas the amount of confident errors did not vary as a function of delay length across both groups, the amount of not-confident errors increased with longer delay lengths, but this was not significantly stronger in PSZ—a response pattern which would have provided most convincing evidence for a WM maintenance deficit in schizophrenia. We can, however, not fully exclude that this non-significant three-way interaction was due to a lack of power; see also next section.

### Limitations

A limitation of our study, as already discussed above, was the relatively small sample of PSZ, which may have contributed to a lack of significance for example of the three-way interaction of delay length, error type, and group just discussed (see section Not-Confident Errors Represent Failures of WM Maintenance in Schizophrenia) and/or the finding that WM performance reached a minimum at a delay length of 4 s and did not further decrease at a delay length of 6 s in PSZ (see section WM Deficits in Schizophrenia). In order to control for potential confounds due to the heterogeneity of schizophrenia, the patient sample—albeit relatively small— was homogeneous in terms of diagnoses including mostly patients with paranoid schizophrenia. This may counteract to a certain degree the danger of false positive or negative results due to the small sample size. However, we think that it is important to replicate the present findings in larger samples.

In this sample, we replicated the increased rate of confident yet incorrect responses in the DRT in PSZ compared to HC which had been reported before (43, 44, 55). However, the increased amount of not-confident errors in PSZ compared to HC observed in this study is in contrast to the primary encoding deficit suggested by previous studies (2, 43, 44), and the reason for this discrepancy is not clear. Besides the sample size, task-related factors and/or differences in clinical characteristics of the patient samples (e.g., degree of positive symptoms and disease duration) might have influenced the severity of encoding and maintenance deficits reported in different studies. However, neither the severity of current positive nor the severity of negative symptoms was related to any of the WM performance measures, a finding that is consistent with previous evidence (11, 43, 44), suggesting that WM deficits in schizophrenia are stable and exist across clinical states. Moreover, WM encoding and maintenance deficits might not be independent from the generalized cognitive deficit observed in schizophrenia (60, 61). Therefore, future studies are needed to systematically investigate the severity of maintenance vs. encoding deficits across different disease phases as well as their relations to the generalized cognitive deficit.

Another potential limitation of this study concerns possible effects of medication. To assess the influence of medication, we correlated task performance with CPE dose. Only d′-correlated with CPE (see Supplementary Material 2, Table S6). However, group differences in d′ remained significant when taking daily CPE dose into account by including it as a covariate in the analyses. Importantly, we have previously demonstrated that unaffected and unmedicated first-degree relatives of PSZ also show an increased rate of false memories when performing similar spatial DRTs (43, 44). Therefore, it seems not likely that the WM deficit in terms of confident errors (false memories) and not-confident errors observed in the present sample of patients can be explained solely in terms of medication.

Another caveat was the difference in education between HC and PSZ. Because cognitive abilities and memory could be strengthened with education, it was important to rule out the possibility that the observed deficits in WM were merely a consequence of group differences in education. Therefore, we re-ran the analyses with a subset of PSZ and HC matched on years of education (see Supplementary Material 1.2). The results were highly comparable to the results found in the analyses of the entire sample, indicating that the group differences in WM performance were not primarily driven by differences in education.

Finally, signal detection analysis was used to assess differences in overall response strategies between PSZ and HC. Specifically, we compared the response criterion c between groups at different delay lengths. In both groups the response criterion c increased with longer delay lengths. Thus, with longer delays participants' response strategy changed from more liberal to more conservative. Most importantly, PSZ and HC did not differ in this response bias. These findings indicate that PSZ implemented a similar response strategy as HC and do not support the possibility that the patients' deficit was due to differences in an overall response bias between PSZ and HC.

### Implications for neural mechanisms of WM deficits in schizophrenia

The present findings of impairments of WM in schizophrenia at the level of encoding and maintenance processes are consistent with findings from electrophysiological(33–39, 62) and event-related fMRI studies (17, 18, 27, 38, 40, 41, 55) that explicitly examined WM encoding-related and WM maintenance-related neural abnormalities in schizophrenia and in unaffected first-degree relatives of PSZ (27, 40). These studies suggest that a PFC dysfunction—which has been discussed as a core mechanism underlying WM deficits in schizophrenia (1, 5)—contribute not only to impaired WM maintenance but also to impaired WM encoding. Moreover, these studies revealed encoding-specific disturbances in the interplay between PFC and posterior visual-parietal cortex critical for the transfer of perceptual information into WM (18, 27, 40, 41). Several candidate mechanisms for impaired early-stage visual processing and/or higher-level cognitive processes required for WM encoding have been implicated by electrophysiological studies and discussed as primary constraints of WM capacity (33–39). Furthermore, these dysfunctions have been related to the GABAergic and/or glutamatergic prefrontal pathology that is central to WM-related deficits in schizophrenia (20–22, 34, 39, 62–64).

However, linking specific WM-related deficits—such as encoding vs. maintenance deficits—to specific alterations in synaptic transmission in schizophrenia is a challenging task. One promising approach is the development of neurocomputational network models of WM deficits in schizophrenia (7, 8, 65, 66) that provide a mechanistic link between synaptic alterations and behavioral outputs. Such outputs can then be compared to the WM deficits observed in clinical studies. With regard to spatial WM, Cano-Colino and Compte (8) have provided a neurocomputational network model of WM deficits in schizophrenia linking synaptic alterations in prefrontal circuits to specific types of errors in the spatial DRT, which are conceptually very similar to the confident and not-confident errors as defined in our study. Specifically, these authors demonstrated that simulating the schizophrenic state by disinhibition of the network through a global reduction of inhibitory GABAergic transmission and/or a specific reduction of NMDA receptor transmission in interneurons (8), both of which have been identified as neurochemical characteristics of schizophrenia (20, 22, 63), lead to a selective increase of confident errors—i.e., false memory errors. At the neural level, this type of error was predominantly associated with a destabilized spontaneous state, i.e., spontaneous activity representing random spatial locations, which emerged before the target actually appeared in the encoding phase of the task, and that remained high throughout the delay period. This firing pattern is equivalent to persistent activity of the wrong representation as previously described in functional imaging studies in schizophrenia (55) and, most importantly, is consistent with the non-delay-dependent behavioral characterization of confident errors in the present study. We also found that the rate of not-confident errors was increased in PSZ and increased with longer delay lengths, reflecting decreased stability of the WM representation during the delay phase. These observations are also consistent with the predictions of the network model (8) in which not-confident errors were characterized by a decay in the stimulus-specific network activity by the end of the delay when NMDA receptor transmission was globally reduced.

In contrast, the increased amount of confident errors was not consistent with the simulations of another neurocomputational model for WM deficits in schizophrenia developed by Murray (66). This computational model was analogous to the one used by Cano-Colino and Compte (8), however disinhibition in the network was implemented only through a subtle NMDA receptor conductance decrease to inhibitory interneurons. This synaptic manipulation resulted in a stable but broader network activity pattern during the delay period, which was associated at the behavioral level with reduced precision of the WM representation rather than the maintenance of a wrongly encoded location (66). Whereas this network regime can account for WM maintenance deficits observed in schizophrenia (67), it cannot account for the WM encoding deficit (2) as indexed by the increased rate of false memory errors in schizophrenia observed in this and previous behavioral studies (43, 44).

Schizophrenia is a highly heterogeneous disorder and it is possible that WM impairments are caused by distinct functional deficits depending on the subpopulation, disease stage, and/or symptom severity. In addition, different WM modalities (e.g., visual vs. spatial) that are mediated by distinct neurophysiological mechanisms might be differentially disrupted. We suggest that the behavioral paradigm used in the present study—i.e., explicitly comparing confident vs. not-confident error responses—through its interesting link to the behavioral outputs of the discussed computational models, may provide an interesting approach for more directly comparing the neurocomputational simulations with behavioral performance patterns of PSZ.

## Conclusion

Taken together, the present findings suggest across both groups a functional dissociation of confident and not-confident errors in the spatial DRT, associating them with encoding vs. maintenance processes of WM, respectively. Furthermore, by demonstrating increased rates of both confident (i.e., false memory) and not-confident errors in schizophrenia, we provide additional empirical evidence that supports the assumed impairments of WM in schizophrenia at the level of encoding and maintenance processes. Our findings underscore the relevance of distinguishing different error types in the DRT in order to reveal the various aspects of WM dysfunction in schizophrenia. By combining this approach across behavioral, neurophysiological, and possibly also neurocomputational levels of analysis, future studies have the potential to link specific aspects of WM impairment to their underlying neural dysfunctions and thus to provide a better neurocognitive basis for guiding the development of targeted intervention strategies.

## Author contributions

JM and CF developed the study design. Data collection was performed by JM. MS and VO-K organized patient recruitment and assisted in clinical assessments. Data analysis and interpretation were performed by JM and CF. JM and CF wrote the present article. MS and VO-K provided critical revisions. All the authors approved the final version of the manuscript for submission.

### Conflict of interest statement

The authors declare that the research was conducted in the absence of any commercial or financial relationships that could be construed as a potential conflict of interest.

## Footnotes

^1^The Mann-Whitney U test was used because IQ was not normally distributed in PSZ.
