# Supplementary material for: Functional Dissociation of Confident and Not-Confident Errors in the Spatial Delayed Response Task Demonstrates Impairments in Working Memory Encoding and Maintenance in Schizophrenia
Source: Front Psychiatry. 2018 May 29;9:202. doi: 10.3389/fpsyt.2018.00202 (PMC5987160; doi:10.3389/fpsyt.2018.00202)
Supplement: Supplementary file 1 [file Data_Sheet_1.docx]

Supplementary Material

Functional Dissociation of Confident and Not-Confident Errors in the Spatial Delayed Response Task Demonstrates Impairments in Working Memory Encoding and Maintenance in Schizophrenia

**Jutta S. Mayer*, Michael Stäblein, Viola Oertel-Knöchel, Christian J. Fiebach**

**Correspondence:** Jutta S. Mayer: [jutta.mayer@kgu.de](mailto:jutta.mayer@kgu.de)

# Supplementary Data

## Description of the fulfillment of the ANOVA’s assumptions for the groups studied

For each group and each factor level, dependent variables were assessed for normality using the Shapiro-Wilk-Test. Homogeneity of error variances across groups was assessed using Levene’s test and homogeneity of covariances were assessed by Box’s test. In case of violation of any of these assumptions, we reported additionally re-analyses of the respective effects with non-parametric tests The Mann-Whitney U test was used to assess group effects and Friedman or Wilcoxon tests were used to assess effects of delay length. Greenhouse–Geisser corrected *p*-values were reported in cases where ANOVA sphericity assumptions were violated, which were determined with a Mauchly's test for sphericity (*p* < 0.05).

### Accuracy and RTs

# 2 x 4 repeated-measures ANOVA were conducted to test for differences in accuracy and RTs as a function of delay length (1 s, 2 s, 4 s, 6 s) and group (PSZ vs. HC); see Section Results: Accuracy and Reaction Times in the main manuscript.

# Accuracy was normally distributed for both groups and all delay lengths as assessed by the Shapiro-Wilk-Test (see Table S1). In addition, there was homogeneity of covariances across groups as assessed by Box’s test (Box’s *M* = 9.08, *F* = 0.82, *p* = .61). For all delay lengths, errors variances were equal across groups as assessed by Levene’s test (see Table S2). For PSZ, RTs were normally distributed for all delay lengths. For HC, RTs were not normally distributed (see Table S1). Covariance matrices were not equal for HC and PSZ (Box’s *M* = 30.95, *F* = 2.80, *p* < .01). There was homogeneity of error variances across groups for all delay lengths as assessed by Levene’s test (see Table S2).

# Because RTs were not normally distributed for HC, we conducted additional non-parametric tests. Because the ANOVA revealed a significant group effect, we tested for a group difference in RTs using the Mann-Whitney U test and found a significant effect (*U* = 100.0, *p* < .001). Furthermore, the ANOVA revealed a significant effect of delay length on RTs which was also revealed by the Friedman test (χ^2^(3, N = 48) = 19.33, *p* < .001). We also assessed the interaction between the factors group and delay length with a non-parametric approach. For each participant, we first computed the differences in RTs between delay lengths of 6s and 1s. We then tested whether these difference values differed between groups using the Mann-Whitney U test. Consistent with the non-significant interaction effect revealed by the ANOVA, differences in RTs between delay lengths of 6s and 1s did not differ between groups (*U* = 244, *p* = .36). Taken together, results of parametric and non-parametric tests on RTs were comparable.

### Confident and not-confident error responses

A 2 x 4 x 2 repeated-measures ANOVA was conducted to test for differences in the percentage of type of error response (confident vs. not confident) as a function of delay length (1 s, 2 s, 4 s, 6 s) and group (PSZ vs. HC); see Section Results: Confident and Not-Confident Error Responses in the main manuscript.

# For PSZ, percentages of confident errors were normally distributed for all delay lengths. For HC, percentages of confident errors were normally distributed except for delay length of 1s. Percentages of not-confident errors were normally distributed except for delay length of 1s for PSZ (see results on the Shapiro-Wilk-Test, Table S1). For HC, percentages of not-confident errors were normally distributed only for delay lengths of 6s (see Table S1). Covariance matrices were equal for HC and PSZ as assessed by Box’s test (Box’s *M* = 55.84, *F* = 1.26, *p* = .14). For the percentages of confident errors, error variances were not equal across groups except for delay length of 6s. For the percentages of not-confident errors, error variances were not equal across groups except for delay length of 2s (see Table S2).

# The ANOVA revealed a significant interaction between the factors error type and delay length. Because the assumptions of normality and homogeneity of error variances across groups were violated for some conditions, we assessed this interaction effect with a non-parametric approach. For each participant, we first computed the difference between delay lengths of 6s and 1s for the percentages of confident and not-confident errors. We then tested whether the difference values were different for confident and not-confident errors using the Wilcoxon test. The Wilcoxon test revealed a significant effect (*Z* = -2.6, *p* < .01), a result that was consistent with the significant interaction between the factors error type and delay length revealed by the ANOVA. Furthermore, we assessed the effect of delay length separately on the percentage of confident and not-confident errors across groups using post-hoc Friedman tests. The results revealed a significant effect of delay length on the percentage of not-confident responses (χ^2^(3, N = 48) = 28.08, *p* < .001) but not on the percentage of confident errors (χ^2^(3, N = 48) = 0.71, *p* = .87). Taken together, these findings were comparable to the results revealed by the ANOVA.

# We also used a non-parametric approach to assess the group x error type interaction which was non-significant in the ANOVA. Differences between errors types (i.e. percentage of confident errors minus percentage of not-confident errors) did not differ between groups (*U* = 253.0, *p* = .47). Non-parametric post-hoc tests revealed significant group differences in the percentage of confident errors (*U* = 195.5, *p* < .01) and the percentage of not-confident errors (*U* = 155.0, *p* < .01.). These findings were comparable to the results revealed by the ANOVA.

### Sensitivity (d') and response criterion (c)

# 2 x 4 repeated-measures ANOVAs were conducted to test for differences in sensitivity and the response criterion as a function of delay length (1 s, 2 s, 4 s, 6 s) and group (PSZ vs. HC); see Section Results: Sensitivity (d') and Response Criterion (c) in the main manuscript.

# The sensitivity index d' was normally distributed for both groups except for delay length of 4s as assessed by the Shapiro-Wilk-Test (see Table S1). There was homogeneity of covariances across groups (Box’s *M* = 8.29, *F* = 0.75, *p* = .68). For all delay lengths, errors variances were equal across groups as assessed by Levene’s test (see Table S2).

# Because the assumption of normality was violated for some conditions, we conducted additional non-parametric tests. The Mann-Whitney U test revealed a significant group difference in the sensitivity index d' (*U* = 68.0, *p* < .001.). In addition, there was a significant effect of delay length on the sensitivity index d' as revealed by the Friedman test (χ*^2^*(3, N = 48) = 24.54, *p* < .001). These effects were comparable to the effects of group and delay length on d' revealed by the ANOVA. Because the ANOVA also revealed a significant interaction between the factors group and delay length, we also assessed this interaction effect with a non-parametric approach. For each participant, we computed the difference in d' between delay lengths of 6s and 1s and tested whether difference values differed between groups. As indicated by the Mann-Whitney U test, the difference values did not differ between groups (*U* = 259.0, *p* = .55.). Thus, parametric and non-parametric approaches were comparable with regard to the main effects of group and delay lengths but not with regard to the interaction effect.

# For PSZ, the response criterion c was normally distributed except for delay length of 4s. For HC, the response criterion c was normally distributed except for delay lengths of 4s and 6s (see results on the Shapiro-Wilk-Test, Table S1). There was homogeneity of covariances across groups (Box’s *M* = 18.05, *F* = 1.63, *p* = .09) as assessed by Box’s test. Errors variances were equal across groups for all delay lengths except for delay length of 1s as assessed by Levene’s test (see Table S2).

# Because the assumptions of normality and homogeneity of error variances across groups were violated for some conditions, we conducted additional non-parametric tests. The ANOVA only revealed a significant effect of delay length on c. This effect was also tested with the Friedman test and was significant (χ*^2^*(3, N = 48) = 30.52, *p* < .001). There was no significant group effect (*U* = 280.0, *p* = .87). In addition, differences in the response criterion c between delay lengths of 6s and 1s did not differ between groups (*U* = 253.0, *p* = .47). Therefore, the results of parametric and non-parametric tests were comparable.

## Testing for effects of delay length, error type and group in a subsample of participants matched on education

# Years of education were significantly higher in HC than PSZ. It could be argued that cognitive abilities and memory could be strengthened with education. To rule out the possibility that the differences in means observed between groups were driven by differences in years of education, we therefore reran the analyses on the WM performance indices (RT, accuracy, d', c) and the percentage of specific error types (i.e., incorrect/ confident vs. incorrect/ not-confident responses) in subgroups of PSZ and HC who were matched on the number of years of education (see Table S3). We individually matched each patient with a HC allowing a difference of only two years. Using this criterion, 12 PSZ and HC were fully matched on years of education, 5 pairs of PSZ and HC differed by one year and 1 pair of PSZ and HC differed by two years, yielding two subsamples of 18 participants each. 4 HCs (> 20 years of education) and 4 patients (years of education between 10 and 13) were excluded using this stringent criterion. The fulfillment of the ANOVA’s assumptions was tested and non-parametric tests were conducted in case these assumptions were violated as described in 1.1.

### Accuracy and RTs

# 2 x 4 repeated-measures ANOVAs were conducted to test for differences in accuracy and RTs as a function of delay length (1 s, 2 s, 4 s, 6 s) and group (PSZ vs. HC)

# Accuracy was normally distributed for both groups and all delay lengths as assessed by the Shapiro-Wilk-Test (see Table S4). There was homogeneity of covariances across groups as assessed by Box’s test (Box’s *M* = 3.77, *F* = 0.33, *p* = .97). For all delay lengths, errors variances were equal across groups as assessed by Levene’s test (see Table S5).

# Response accuracy was significantly lower in PSZ compared to HC [*F*(1,34) = 17.50, p < .001, *η^2^* = .34]. Response accuracy decreased with longer delay lengths [*F*(3, 102) = 7.86, p < .001, *η^2^* = .19] and was explained best by a linear trend [*F*(1,34) = 23.15, *p* < .001, *η^2^* = .41; quadratic trend, *F*(1,34) = 1.78, *p* = .19; cubic trend, *F*(1,34) = 0.13, *p* = .72]. As indicated by a significant interaction between the factors delay length and group [*F*(3,102) = 4.54, *p* < .01, *η^2^* = .12], this delay-dependent decrease in accuracy was stronger in PSZ [*F*(3, 51) = 7.73, *p* < .001, *η^2^* = .31] than HC [*F*(3, 51) = 4.47, *p* < .01, *η^2^* = .21]. These findings were comparable to the results revealed in the non-matched groups.

# RTs were normally distributed for both groups and all delay lengths as assessed by the Shapiro-Wilk-Test (see Table S4). Covariance matrices were not equal for HC and PSZ (Box’s *M* = 43.75, *F* = 3.81, *p* < .001). There was homogeneity of error variances across groups only for delay lengths 2s and 6s as assessed by Levene’s test (see Table S5).

# RTs were significantly longer in PSZ compared to HC [*F*(1,34) = 24.27, *p* < .001, *η^2^* = .42], a result that was consistent with the group effect revealed by the ANOVA in the entire sample. In contrast to the finding from the ANOVA in the entire sample, RTs did not increase with longer delay lengths [non-significant effect of delay length: *F*(2.40, 81.43) = 1.04, *p* = .37]. The interaction between the factors group and delay length was not significant [*F*(2.40, 81.43) = 0.24, *p* =.87], which again was consistent with the finding in the entire sample.

# Because the assumptions of homogeneity of covariance matrices and homogeneity of error variances across groups were violated for some conditions, we conducted additional non-parametric tests. The Mann-Whitney U test revealed a significant group difference in RTs (*U* = 33, *p* < .001). In addition, the Friedman test revealed that RTs increased with longer delay lengths (χ^2^(3, N = 36 = 12.83, *p* < .01). We also assessed the interaction between the factors group and delay length with a non-parametric approach. For each participant, we first computed the differences in RTs between delay lengths of 6s and 1s. We then tested whether these difference values differed between groups using the Mann-Whitney U test. Differences in RTs between delay lengths of 6s and 1s did not differ between groups (*U* = 135, *p* = .39). These findings were largely comparable with the findings revealed by the ANOVA.

# Overall, the findings observed in the matched groups do not suggest that the effects on accuracy and RTs observed in the non-matched samples were largely driven by group differences in education.

### Confident and not-confident error responses

# A 2 x 4 x 2 repeated-measures ANOVA was conducted to test for differences in the percentage of type of error response (confident vs. not confident) as a function of delay length (1 s, 2 s, 4 s, 6 s) and group (PSZ vs. HC).

# For both groups, the percentages of confident errors were normally distributed for all delay lengths (see Table S4). Percentages of not-confident errors were also normally distributed for all delay lengths for PSZ. For HC, percentages of not-confident errors were normally distributed only for delay lengths of 6s (see Table S4). Covariance matrices were equal for HC and PSZ as assessed by Box’s test (Box’s *M* = 59.81, *F* = 1.24, *p* = .16). For the percentages of confident errors, error variances were equal across groups for all delay lengths. For the percentages of not-confident errors, error variances were not equal across groups for delay lengths of 1s and 4s (see Table S5).

# The ANOVA revealed a significant interaction between the factors error type and delay length [*F*(3,102) = 5.14, *p* < .01, *η^2^* = .13]. Across both groups the percentage of incorrect/ not-confident responses increased with longer delay lengths [*F*(3,105) = 10.36, *p* < .001, *η^2^* = .23] and was explained best by a linear trend [*F*(1,35) = 25.98, *p* < .001, *η^2^* = .44; quadratic trend, *F*(1,35) = 1.69, *p* = .20; cubic trend, *F*(1,35) = 0.04, *p* = .84]. In contrast, the percentage of incorrect/ confident responses did not vary between delay lengths [*F*(3,105) = 0.36, *p* = .78]. These findings were comparable to the results revealed in the non-matched groups.

Because the assumptions of normality and homogeneity of error variances across groups were violated for some conditions, we assessed this interaction effect with a non-parametric approach. For each participant, we first computed the difference between delay length 6s and 1s for the percentages of confident and not-confident errors. We then tested whether the difference values were different for confident and not-confident errors using the Wilcoxon test. The Wilcoxon test revealed a significant effect (*Z* = -2.77, *p* < .01), a result that was consistent with the significant interaction between the factors error type and delay length as revealed by the ANOVA. Furthermore, we assessed the effect of delay length separately on the percentage of confident and not-confident errors across groups using post-hoc Friedman tests. Friedman tests revealed a significant effect of delay length on the percentage of not-confident responses (χ^2^(3, N = 36) = 23.97, *p* < .001) but not on the percentage of confident errors (χ^2^(3, N = 36) = 0.35, *p* = .95). Thus, findings from parametric and non-parametric tests were comparable.

# When comparing the WM deficit of PSZ with performance in the control group, the ANOVA revealed a significant group effect [*F*(1,34) = 17.50, *p* < .001, *η^2^* = .34 (see also results for response accuracy in the previous section)], but no interaction between the factors group and error type [*F*(1,34) = 0.18, *p* = .68]. Thus, both, the percentage of incorrect/ confident responses [=false memory errors, 17.02% (SD = 9.35) vs. 11.21 % (SD = 7.19) for PSZ and HC, respectively, *t*(34) = -2.09, p < .05] and the percentage of incorrect/ not-confident responses [15.12% (SD = 8.70) vs. 7.47% (SD = 5.24) for PSZ and HC, respectively, *t*(34) = -3.20, *p* < .01], were increased in PSZ compared to HC. These findings were comparable to the results revealed in the entire sample.

# We also used a non-parametric approach to assess the group x error type interaction which was non-significant in the ANOVA. Differences between errors types (i.e. percentage of confident errors minus percentage of not-confident errors) did not differ between groups (*U* = 135.5, *p* = .40). Non-parametric post-hoc tests revealed significant group differences in the percentage of confident errors (*U* = 98.5, *p* < .05) and the percentage of not-confident errors (*U* = 71.5, *p* < .01.). These findings were comparable to the results revealed by the ANOVA.

# Finally, the three-way interaction between the factors delay length, response type, and group was not significant [*F*(3,102) = 1.08, *p* = .36], indicating that in the present sample the differential effect of delay length on the amount of confident errors (i.e., not delay-dependent; false memory errors) and not-confident errors (i.e., delay-dependent) did not significantly differ between groups. Across types of errors, however, the effect of delay length was stronger in PSZ compared to HC [significant interaction between the factors delay length and group, *F*(3,102) = 4.54, *p* < .01, *η^2^* = .12, see also response accuracy]. These findings were comparable to the results revealed in the entire sample.

# Taken together, the findings from matched subsamples did not suggest that group differences in the amount of confident and not-confident errors observed in the non-matched samples were a consequence of group differences in education.

### Sensitivity (d') and response criterion (c)

# 2 x 4 repeated-measures ANOVAs were conducted to test for differences in sensitivity and the response criterion as a function of delay length (1 s, 2 s, 4 s, 6 s) and group (PSZ vs. HC).

# The sensitivity index d' was normally distributed for all conditions except for PSZ for delay length of 4s as assessed by the Shapiro-Wilk-Test (see Table S4). There was homogeneity of covariances across groups (Box’s *M* = 10.08, *F* = 0.88, *p* = .55). For all delay lengths, errors variances were equal across groups as assessed by Levene’s test (see Table S5).

# The sensitivity of the discrimination between target and non-target position in the DRT was significantly lower in PSZ compared to HC [*F*(1, 34) = 18.07, *p* < .001, *η^2^* = .35]. Furthermore, d' decreased with increasing delay lengths [*F*(3, 102) = 3.58, *p* < .05, *η^2^* = .10], and was explained best by a linear trend [*F*(1,34) = 15.73, *p* < .001, *η^2^* = .32; quadratic trend, *F*(1,34) = 0.88, *p* = .35; cubic trend, *F*(1,34) = 0.04, *p* = .84]. As indicated by a significant interaction between the factors delay length and group [*F*(3, 102) = 4.04, *p* < .01, *η^2^* = .11], this delay-dependent decrease was significant in PSZ [*F*(3, 51) = 6.796, *p* < .001, *η^2^* = .29] but not in HC [*F*(3, 51) = 1.83, *p* = .15]. These findings were comparable to the results revealed in the non-matched groups and therefore did not suggest that group differences in the sensitivity index d' observed in the entire sample were a consequence of group differences in education.

# Because the assumption of normality was violated for some conditions, we conducted additional non-parametric tests. The Mann-Whitney U test revealed a significant group effect on d' (*U* = 49.0, *p* < .001.). In addition, there was a significant effect of delay length on the sensitivity index d' as revealed by the Friedman test (χ*^2^*(3, N = 36) = 15.12, *p* < .01). However, differences in d' between delay lengths of 6s and 1s did not differ between groups (*U* = 146.0, *p* = .61.). Thus, parametric and non-parametric approaches revealed comparable results for main effects of group and delay length but not with regard to the interaction effect.

# The response criterion c was normally distributed for all conditions except for PSZ for delay length of 4s as assessed by the Shapiro-Wilk-Test (see Table S4). There was homogeneity of covariances across groups (Box’s *M* = 14.57, *F* = 1.27, *p* = .24) as assessed by Box’s test. Errors variances were equal across groups for all delay lengths except for delay length 1s as assessed by Levene’s test (see Table S5).

# The response criterion c increased with longer delay lengths [*F*(2.55, 86.88) = 9.66, *p* < .001, *η^2^* = .22] and was explained best by a linear trend [*F*(1, 34) = 23.05, *p* < .001, *η^2^* = .40; quadratic trend, *F*(1,34) = 0.007, *p* = .935; cubic trend, *F*(1,34) = 1.09, *p* = .30]. However, there was neither a significant group effect [*F*(1, 34) = 0.005, *p* = .946], nor a significant interaction between the factors delay length and group [*F*(2.55, 86.88) = 0.63, *p* =.57] on the response criterion c. These findings were comparable to the results revealed in the entire sample.

# Because the assumptions of normality and homogeneity of error variances across groups were violated for some conditions, we conducted additional non-parametric tests. The Friedman test revealed a significant effect of delay length on the response criterion c (χ*^2^*(3, N = 36) = 19.57, *p* < .001). There was no significant group difference as indicated by the Mann-Whitney U test (*U* = 150.0, *p* = .72). In addition, differences in the response criterion c between delay lengths of 6s and 1s did not differ between groups (*U* = 155.0, *p* = .83). These findings were consistent with the results revealed by the ANOVA.

# Supplementary Tables

**Table S1. Results on normality testing**

|  |  | | **Accuracy** | | | **Response Times** | | |
| --- | --- | --- | --- | --- | --- | --- | --- | --- |
| **Delay** | **Group** | | Statistic | df | Sig. | Statistic | df | Sig. |
| 1s | | HC | .929 | 24 | .095 | .812 | 24 | .000 |
|  |  | PSZ | .947 | 24 | .228 | .937 | 24 | .138 |
| 2s | | HC | .978 | 24 | .859 | .883 | 24 | .009 |
|  |  | PSZ | .936 | 24 | .136 | .955 | 24 | .350 |
| 4s | | HC | .979 | 24 | .877 | .857 | 24 | .003 |
|  |  | PSZ | .943 | 24 | .193 | .927 | 24 | .082 |
| 6s | | HC | .971 | 24 | .685 | .899 | 24 | .020 |
|  |  | PSZ | .956 | 24 | .364 | .931 | 24 | .103 |
|  | | |  | | |  | | |
|  |  | | **Confident errors** | | | **Not-confident errors** | | |
| **Delay** | **Group** | | Statistic | df | Sig. | Statistic | df | Sig. |
| 1s | | HC | .905 | 24 | .027 | .870 | 24 | .005 |
|  |  | PSZ | .954 | 24 | .330 | .908 | 24 | .032 |
| 2s | | HC | .929 | 24 | .090 | .900 | 24 | .022 |
|  |  | PSZ | .956 | 24 | .370 | .952 | 24 | .297 |
| 4s | | HC | .929 | 24 | .093 | .837 | 24 | .001 |
|  |  | PSZ | .955 | 24 | .349 | .957 | 24 | .388 |
| 6s | | HC | .935 | 24 | .128 | .956 | 24 | .357 |
|  |  | PSZ | .946 | 24 | .222 | .921 | 24 | .061 |
|  | | |  | | |  | | |
|  | | | **Sensitivity d'** | | | **Response criterion c** | | |
| **Delay** | **Group** | | Statistic | df | Sig. | Statistic | df | Sig. |
| 1s | | HC | .932 | 24 | .106 | .972 | 24 | .714 |
|  |  | PSZ | .958 | 24 | .396 | .970 | 24 | .665 |
| 2s | | HC | .982 | 24 | .932 | .968 | 24 | .628 |
|  |  | PSZ | .926 | 24 | .078 | .981 | 24 | .906 |
| 4s | | HC | .902 | 24 | .024 | .904 | 24 | .026 |
|  |  | PSZ | .866 | 24 | .004 | .881 | 24 | .009 |
| 6s | | HC | .946 | 24 | .223 | .913 | 24 | .041 |
|  |  | PSZ | .939 | 24 | .154 | .970 | 24 | .660 |

*Note.* HC = healthy controls; PSZ = patients with schizophrenia

**Table S2. Homogeneity of the error variances across groups**

| **Delay** | **Accuracy** | **Reaction times** |
| --- | --- | --- |
| 1s | *F*(1,46) = 0.05, *p* = .82 | *F*(1,46) = 0.91, *p* = .35 |
| 2s | *F*(1,46) = 1.30, *p* = .26 | *F*(1,46) = 0.04, *p* = .84 |
| 4s | *F*(1,46) = 0.04, *p* = .84 | *F*(1,46) = 1.09, *p* = .30 |
| 6s | *F*(1,46) = 0.002, *p* = .96 | *F*(1,46) = 0.50, *p* = .48 |
| **Delay** | **Confident errors** | **Not-confident errors** |
| 1s | *F*(1,46) = 4.32, *p* < .05 | *F*(1,46) = 8.20, *p* < .01 |
| 2s | *F*(1,46) = 4.46, *p* < .05 | *F*(1,46) = 2.95, *p* = .09 |
| 4s | *F*(1,46) = 5.31, *p* < .05 | *F*(1,46) = 7.89, *p* < .01 |
| 6s | *F*(1,46) = 0.30, *p* = .59 | *F*(1,46) = 12.02, *p* < .01 |
| **Delay** | **Sensitivity d'** | **Response criterion c** |
| 1s | *F*(1,46) = 0.35, *p* = .56 | *F*(1,46) = 6.73, *p* < .05 |
| 2s | *F*(1,46) = 0.80, *p* = .38 | *F*(1,46) = 2.51, *p* = .12 |
| 4s | *F*(1,46) = 0.20, *p* = .66 | *F*(1,46) = 2.30, *p* = .14 |
| 6s | *F*(1,46) = 0.92, *p* = .34 | *F*(1,46) = 1.92, *p* = .17 |

**Table S3. Demographic and clinical information for subgroups matched on education**

|  | **PSZ**  **n = 18** | **HC**  **n = 18** | **Statistic** |
| --- | --- | --- | --- |
| Age  Range | 42.94 (11.71)  21-59 | 39.94 (10.28)  24-60 | *t*(34) = -0.82, *p* = .42 |
| Female/male | 5/13 | 8/10 | *χ^2^*(1, N = 36) = 1.08, *p* = .30 |
| Race (Caucasian)  Handedness (right/left) | 18  18/0 | 18  18/0 |  |
| Years of education | 15.72 (2.05) | 16.11 (2.17) | *t*(34) = 0.55, p = .58 |
| Years of education  Mother^a^  Father^b^ | 13.00 (4.09)  12.00 (4.70) | 12.86 (4.35)  12.07 (3.67) | *t*(28) = 0.05, *p* = .96  *t*(27) = -0.09, *p* = .93 |
| IQ^c^ | 106.11 (11.99) | 108.83 (11.65) | *U* = 137.5, *p* = .44 |
| CPE, mg/day | 511.47 (261.12) | n/a |  |
| Years of illness | 11.81 (8.64) | n/a |  |
| PANSS – positive  SAPS | 15.17 (4.48)  21.52 | n/a |  |
| PANSS – negative  SANS | 15.94 (6.76) 25.10 | n/a |  |
| PANSS - general | 31.78 (8.36) | n/a |  |

*Note.*  ^a^Three patients and four controls could not provide this information.

^b^Two patients and four controls could not provide this information. Missing data were handled by excluding cases analysis by analysis. ^c^IQ was not normally distributed for PSZ (Shapiro-Wilk-Test, *p* < .01). We therefore report as statistics *U* from a Mann-Whitney test. *CPE =* current daily Chlorpromazine equivalent, PANSS = Positive and Negative Symptom Scale, SAPS = Scale for the Assessment of Positive Symptoms, SANS = Scale for the Assessment of Negative Symptoms.

**Table S4. Results on normality testing in matched subgroups**

|  |  | | **Accuracy** | | | **Response Times** | | |
| --- | --- | --- | --- | --- | --- | --- | --- | --- |
| **Delay** | **Group** | | Statistic | df | Sig. | Statistic | df | Sig. |
| 1s | | HC | .927 | 18 | .169 | .953 | 18 | .472 |
|  |  | PSZ | .955 | 18 | .516 | .912 | 18 | .093 |
| 2s | | HC | .965 | 18 | .696 | .948 | 18 | .401 |
|  |  | PSZ | .942 | 18 | .309 | .956 | 18 | .525 |
| 4s | | HC | .969 | 18 | .785 | .968 | 18 | .755 |
|  |  | PSZ | .960 | 18 | .608 | .899 | 18 | .056 |
| 6s | | HC | .972 | 18 | .834 | .962 | 18 | .638 |
|  |  | PSZ | .971 | 18 | .814 | .906 | 18 | .074 |
|  | | |  | | |  | | |
|  |  | | **Confident errors** | | | **Not-confident errors** | | |
| **Delay** | **Group** | | Statistic | df | Sig. | Statistic | df | Sig. |
| 1s | | HC | .903 | 18 | .065 | ,851 | 18 | .009 |
|  |  | PSZ | .964 | 18 | .682 | ,913 | 18 | .097 |
| 2s | | HC | .905 | 18 | .072 | ,883 | 18 | .030 |
|  |  | PSZ | .950 | 18 | .427 | ,950 | 18 | .427 |
| 4s | | HC | .905 | 18 | .069 | ,786 | 18 | .001 |
|  |  | PSZ | .921 | 18 | .135 | ,968 | 18 | .761 |
| 6s | | HC | .920 | 18 | .128 | ,948 | 18 | .400 |
|  |  | PSZ | .935 | 18 | .237 | ,913 | 18 | .098 |
|  | | |  | | |  | | |
|  | | | **Sensitivity d'** | | | **Response criterion c** | | |
| **Delay** | **Group** | | Statistic | df | Sig. | Statistic | df | Sig. |
| 1s | | HC | .914 | 18 | .100 | .978 | 18 | .924 |
|  |  | PSZ | .972 | 18 | .829 | .955 | 18 | .513 |
| 2s | | HC | .966 | 18 | .715 | .956 | 18 | .532 |
|  |  | PSZ | .935 | 18 | .236 | .973 | 18 | .857 |
| 4s | | HC | .929 | 18 | .190 | .931 | 18 | .202 |
|  |  | PSZ | .841 | 18 | .006 | .849 | 18 | .008 |
| 6s | | HC | .945 | 18 | .356 | .898 | 18 | .054 |
|  |  | PSZ | .954 | 18 | .490 | .956 | 18 | .521 |

*Note.* HC = healthy controls; PSZ = patients with schizophrenia

**Table S5. Homogeneity of the error variances across matched subgroups**

| **Delay** | **Accuracy** | **Reaction times** |
| --- | --- | --- |
| 1s | *F*(1,34) = 0.04, *p* = .84 | *F* (1,34) = 4.82, *p* < .05 |
| 2s | *F*(1,34) = 2.12, *p* = .15 | *F* (1,34) = 0.46, *p* = .50 |
| 4s | *F* (1,34) = 0.20, *p* = .66 | *F* (1,34) = 5.97, *p* < .05 |
| 6s | *F* (1,34) = 0.03, *p* = .86 | *F* (1,34) = 1.13, *p* = .30 |
| **Delay** | **Confident errors** | **Not-confident errors** |
| 1s | *F* (1,34) = 1.61, *p* = .21 | *F* (1,34) = 6.72, *p* < .05 |
| 2s | *F* (1,34) = 0.90, *p* = .35 | *F* (1,34) = 1.58, *p* = .22 |
| 4s | *F* (1,34) = 3.10, *p* = .09 | *F* (1,34) = 4.22, *p* < .05 |
| 6s | *F* (1,34) = 0.11, *p* = .74 | *F* (1,34) = 3.76, *p* = .06 |
| **Delay** | **Sensitivity d'** | **Response criterion c** |
| 1s | *F* (1,34) = 0.17, *p* = .68 | *F* (1,34) = 5.14, *p* = .03 |
| 2s | *F* (1,34) = 1.12, *p* = .30 | *F* (1,34) = 1.93, *p* = .17 |
| 4s | *F* (1,34) = 0.66, *p* = .42 | *F* (1,34) = 0.91, *p* = .35 |
| 6s | *F* (1,34) = 1.26, *p* = .27 | *F* (1,34) = 0.41, *p* = .53 |

**Table S6. Correlations with medication status**

|  | | **Accuracy** | | **RT** | **incorrect/ confident** | **incorrect/ not-confident** | **d'** | **c** |
| --- | --- | --- | --- | --- | --- | --- | --- | --- |
| **CPE** | Pearson Correlation | | .322 | -.344 | -.209 | -.106 | .420 | -.212 |
|  | Sig. (2-tailed) | | .134 | .107 | .338 | .632 | .046 | .331 |
|  | N | | 23 | 23 | 23 | 23 | 23 | 23 |

*Note. CPE =* current daily Chlorpromazine equivalent. Because CPEs were not available for Flupentixol and Amisulpride, data of one patient treated with these drugs were not included in correlational analyses.

**Table S7. Correlations with symptom severity**

|  | | **Accuracy** | | **RT** | **incorrect/ confident** | **incorrect/ not-confident** | **d'** | **c** |
| --- | --- | --- | --- | --- | --- | --- | --- | --- |
| **PANSS positive** | Pearson Correlation | | -.177 | -.190 | -.066 | .248 | -.153 | .118 |
|  | Sig. (2-tailed) | | .408 | .373 | .759 | .242 | .477 | .582 |
|  | N | | 24 | 24 | 24 | 24 | 24 | 24 |
| **PANSS negative** | Pearson Correlation | | .174 | .179 | -.224 | .077 | .248 | -.109 |
|  | Sig. (2-tailed) | | .416 | .402 | .292 | .719 | .242 | .613 |
|  | N | | 24 | 24 | 24 | 24 | 24 | 24 |
| **PANSS general** | Pearson Correlation | | .114 | .047 | -.206 | .116 | .169 | -.145 |
|  | Sig. (2-tailed) | | .594 | .829 | .333 | .589 | .430 | .499 |
|  | N | | 24 | 24 | 24 | 24 | 24 | 24 |

*Note.* PANSS = Positive and Negative Symptom Scale.
